# Therapeutic efficacy of artemether-lumefantrine in the treatment of uncomplicated *Plasmodium falciparum* malaria in Ethiopia: a systematic review and meta-analysis

**DOI:** 10.1186/s40249-017-0372-5

**Published:** 2017-11-15

**Authors:** Mohammed Biset Ayalew

**Affiliations:** Department of Clinical Pharmacy, School of Pharmacy, College of Medicine and Health Sciences, Gondar University, Gondar, Ethiopia

**Keywords:** Efficacy, Artemether-lumefantrine, *Plasmodium falciparum*, Ethiopia, Meta-analysis

## Abstract

**Background:**

As Ethiopia is one of the sub-Saharan countries with a great burden of malaria the effectiveness of first line anti-malarial drugs is the major concern. The aim of this study was to synthesize the available evidence on the efficacy of artemether-lumefantrine in the treatment of uncomplicated *Plasmodium falciparum* malaria in Ethiopia. This was done by performing a meta-analysis of recent studies conducted in the country on this topic.

**Methods:**

Studies published between January 2010 and January 2017 that reported on the efficacy of artemether-lumefantrine in the treatment of *P. falciparum* malaria in Ethiopian patients were searched for using the PubMed and Google Scholar databases. Ten prospective single-arm cohort studies that followed patients for 28–42 days were included in this analysis. All of the included studies were deemed to be of high quality.

**Results:**

Ten studies involving 1179 patients that were eligible for meta-analysis were identified. At recruitment, the average parasite count per patient was 1 2981/μl of blood. On the third day of treatment, 96.7% and 98.5% of the study subjects become fever-free and parasite-free, respectively. Based on the per protocol analysis, the cure rate after use of artemether-lumefantrine was 98.2% (polymerase chain reaction corrected) and 97.01% (polymerase chain reaction uncorrected) after 28 days of follow-up. The reinfection rate within 28 days was 1.1% and the recrudescence rate was 1.9%.

**Conclusions:**

This review found that the cure rate for uncomplicated *P. falciparum* malaria using artemether-lumefantrine in Ethiopia is still high enough to recommend the drug as a first-line agent. There should be careful periodic monitoring of the efficacy of this drug, as treatment failure may occur due to resistance, sub-therapeutic levels that may occur due to non-adherence, or inadequate absorption.

**Electronic supplementary material:**

The online version of this article (10.1186/s40249-017-0372-5) contains supplementary material, which is available to authorized users.

## Multilingual abstracts

Please see Additional file 1 for translations of the abstract into the five official working languages of the United Nations.

## Background

Malaria is one of the major public health problems in the world. Its burden is very high in Sub-Saharan Africa, where about 90% of all malaria deaths occur [1]. Each minute, six people in Africa die because of malaria [1, 2]. Malaria is also one of the leading causes of avoidable deaths in pregnant women and children in developing countries [2].

Malaria is an important communicable disease affecting large numbers of people in Ethiopia. About 68% of the Ethiopian population lives in high-risk malaria areas [3]. The common malaria-causing species in Ethiopia are *Plasmodium falciparum* and *P. vivax*; roughly 60% of malaria cases in the country are due to the former [4, 5]. An estimated death rate of severe malaria caused by *P. falciparum* in Ethiopia is about 33% in children below 12 years of age and 10% in hospitalized adults [6].

Early diagnosis and timely treatment of malaria with an effective drug is an important strategy to control the disease [7]. However, the emergence of antimalarial drug resistance is one of the challenges towards controlling malaria [1]. In the early 1990s, a chloroquine-resistant *P. falciparum* strain was the main threat to malaria prevention and control in Ethiopia [8]. A failure rate of above 85%for chloroquine was reported in the late 1990s. This event triggered the change of first-line treatment to sulphadoxine-pyrimethamine in 1998 [9, 10]. However, a study conducted on sulphadoxine-pyrimethamine throughout the country in 2003 reported a 72% treatment failure rate [11]. After the occurrence of a large-scale malaria epidemic in 2003 [12] and the concomitant recognition of widespread resistance to sulphadoxine-pyrimethamine [11, 13], the Federal Ministry of Health of Ethiopia adopted artemether-lumefantrine (AL) as the first-line treatment of uncomplicated *P. falciparum* malaria in 2004 [14]. At that time, the baseline efficacy of AL was 99.1%, which showed a treatment failure rate of under 1% [11].The commonly used brand name of a drug containing AL in Ethiopia is coartem.

The World Health Organization (WHO) recommends above 90% parasitological and clinical curative rates for an antimalarial drug to be approved as a first-line agent [15]. There are many studies conducted in different parts of the world that show that AL fulfills this criterion [16–18]. However, regardless of these reports, studies conducted in Thailand, Japan, Kenya, Zanzibar, and Cambodia have indicated the emergency of treatment failure and an evolvement of a drug-resistant parasite against AL [19–24]. An ongoing monitoring of first-line therapies is very important in order to ensure the use of effective drugs and to maintain the progress made to date in decreasing malaria morbidity and mortality [25].

The WHO recommends that the efficacy of first-line antimalarial drugs should be checked at least every two years [15]. As Ethiopia is one of the Sub-Saharan countries with a great burden of malaria, the effectiveness of this first-line drug is of major concern. The purpose of this review was therefore to synthesize the available evidence on the efficacy of AL in the treatment of uncomplicated *P. falciparum* malaria in Ethiopia.

## Methodology

### Search strategy

Literature published between January 2010 and January 2017 that assessed the efficacy of AL in the treatment of uncomplicated *P. falciparum* malaria in Ethiopia was searched for using the online databases PubMed (https://www.ncbi.nlm.nih.gov/pubmed/) and Google Scholar (https://scholar.google.com/).

The search was conducted by combining the following words: ‘efficacy’, ‘therapeutic efficacy’, ‘artemether-lumefantrine’, ‘Coartem’,‘cohort’, ‘in vivo’, ‘*Plasmodium falciparum* malaria’, ‘antimalarial drug’, and ‘Ethiopia’. Selected reference lists of retrieved articles were also searched manually using Google.

### Article selection

The author reviewed all of the identified articles in order to assess eligibility for inclusion based on predefined criteria. Studies done to assess the efficacy of AL on *P. falciparum* mono-infected Ethiopian patients and which were published between January 2010 and January 2017 were included in this review. Studies done on non-human subjects and articles of which the full text could not be obtained were excluded.

### Quality assessment

Validity and methodological quality of all included studies were assessed according to the National Institutes of Health (NIHs) quality assessment tool for observational cohort studies. [26] The tool comprises 14 criteria stated in the form of a question that can be answered as ‘yes’, ‘no’, or ‘not applicable’/‘not reported’/‘cannot determine’. It contains questions that address the sampling, exposure and outcome measurement, the clarity in stating different components of the study, control of confounders, and validity of the study. After carefully evaluating each study against these criteria, each study was classified as being either of ‘good’, ‘fair’, or ‘poor’ quality. ‘Good’ quality was assigned to those studies that fulfilled 85% or more of the criteria. If 30% of the criteria or under was not fulfilled, the study was considered to be of poor quality.

### Data extraction

Relevant information such as study design, study setting, follow-up period, sample size, baseline characteristics of study subjects, fever clearance, parasite clearance, treatment failure, and cure rates were extracted from each article using the well-prepared and piloted data extraction format in the form of table.

### Data analysis

Meta-analysis was performed using Comprehensive Meta-Analysis software version 2.2.064 [27]. Continuous data were presented as mean ± standard deviation. The proportion of the cure rate was calculated based on per protocol and intention-to-treat analysis. The cure rate and 95% confidence intervals were calculated using the DerSimonian-Laird statistical method assuming a random effect model. Since four of the studies did not report the polymerase chain reaction (PCR) corrected cure rate only six studies were included in the PCR corrected cure rate analysis. But all of the10 studies were included in the PCR uncorrected cure rate analysis.

## Results

### Literature search results

**Fig. 1:**
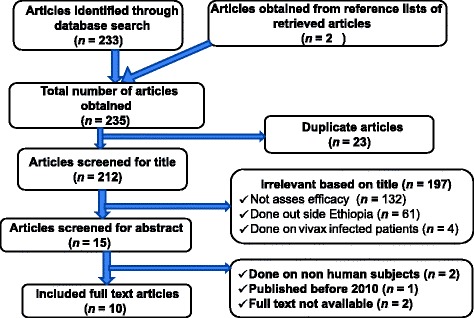
Article selection process

A total of 235 articles were initially retrieved. After excluding duplicates, the titles of 212 articles were assessed and 197 were found to be irrelevant. Abstracts of the remaining 15 articles were checked to determine if they fulfilled the inclusion criteria. Of those, five were rejected: two were done on non-human subjects, one was published before 2010, and the full text was not available for the other two. In the end, 10 articles were found to be suitable to be included in this study. Figure 1 shows the entire article selection process.

### Study characteristics

All 10 studies included in this review were prospective single-arm cohort studies that determined the efficacy of AL in the treatment of uncomplicated *P. falciparum* malaria in Ethiopia.

The studies were conducted in different malarious parts of the country (north, south, east, and central Ethiopia). The WHO guide for surveillance of antimalarial drug efficacy was used in all of the studies to select subjects and to conduct the study.

In the majority (8/10, 80%) of the studies, subjects were followed for 28 days, while in the remaining (2/10, 20%) studies, the follow-up period was 42 days. In each study, anywhere from 66 to 384 patients with *P. falciparum* mono-infection were included. Most (7/10, 70%) of the studies followed patients who were older than six months. One study excluded patients who were below one year of age and another excluded children under five years of age. Details of each study are summarized in Table 1.

**Table 1:** Description of individual study characteristics

sr. no	Author (year of publication)	Study design	Study setting	Study period	Follow up	Subjects	
						Sample size	Inclusion for age
1	Mekonnen SK. et al. (2015) [28]	single arm, open label Prospective cohort study	Omo Nada health center in southwestern Ethiopia	Augest-december 2011	28 days	89	> 6 months old
2	Ebstie YA. et al. (2015) [29]	single arm, open label Prospective cohort study	Bahir Dar district, Northwest Ethiopia	March and July 2012	28 day	134	> 5 years old
3	Eshetu T. et al. (2012) [30]	single arm, open label Prospective cohort study	Agaro Health Centre, Jimma Health Centre, Serbo Health Centre, and Asendabo Health Centre	November 2008 and January 2009 and between August and December 2009.	42 day	348	> 1 year
4	Mulu A. et al. (2015) [31]	single arm, open label Prospective cohort study	Kemisie Health Center, Northeast Ethiopia	September, 2012 to May, 2013	28 days	66	> 6 months old
5	Hwang J. et al. (2011) [32]	single arm, open label Prospective cohort study	Bishoftu Malaria Clinic and Bulbula Health Center, Oromia Regional State	October and November 2009	42 days	119	> 6 months old
6	Nega D et al. (2016) [33]	single arm, open label Prospective cohort study	Metehara Health Centre, Eastern Ethiopia	October 2014 to January 2015	28 days	91	≥ 6 months old
7	Getnet G. et al. (2015) [34]	single arm, open label Prospective cohort study	Enfranze Health Centrer, NW ethiopia	January and May 2013	28-day	80	> 6 months old
8	Assefa A. et al. (2010)^17^	single arm, open label Prospective cohort study	Serbo Health Center, Kersa District, SW Ethiopia.	November 2007 and January 2008	28 day	90	NR
9	Kinfu G. et al. (2012) [35]	single arm, open label Prospective cohort study	Tumuga health center Alamata district, Tigrai regional state, North Ethiopia	August–November 2009.	28 days	71	> 6 months old
10	Wudneh F. et al. (2016) [36]	one-arm prospective open label trial	Gendewuha (Metema) Health Center, NW ethiopia	October 2014 to January 2015	28-day	91	> 6 months old

### Methodological quality of included studies

Each article was evaluated against 14 criteria using the NIHs quality assessment tool for observational cohort studies [26]. Fortunately all of the 10 studies included in this review were found to be of good quality.

### Baseline characteristics of the study subjects

The 10 studies included a total of 1179 participants from 14 study sites. The mean age of study participants was 15.8 years. At recruitment, the average parasite count per patient was 12,981/μl of blood and gametocytes were found in 7.7% of patients. Table 2 shows the mean and standard deviations of baseline characteristics of patients included in the 10 studies.

**Table 2:** Mean baseline characteristics of patients with uncomplicated Falciparum malaria

Characteristics	Mean value	Standard deviation
Age	15.8 years	3.4
Weight	37.3 kg	6.3
Temperature	38.2 °C	0.35
Hemoglobin	12.2 mg/dl	0.89
Parasite load	12,981 Parasites/μl	5261
Patients with Gametocyte	7.7%	4.3

### Fever and parasite clearance rate

Fever and parasite clearance was rapid. On the third day of treatment, 96.7% and 98.5% of study subjects become fever-free and parasite-free, respectively. There was also a significant decrease in gametocyte carriage from 7.7% at baseline to 0.4% on the 28th day of treatment. Table 3 shows the overall progress of fever and parasite clearance in the first three days of AL treatment.

**Table 3:** Fever and parasite clearance rate in the first 3 days of treatment

Parameter	Day 1	Day 2	Day 3
Fever clearance	76.8%	95.6%	96.7%
Parasite clearance	68.1%	93.9%	98.5%

Treatment outcome

### Treatment outcome

Of the 1179 subjects included in the 10 studies reviewed, only 27 (2.29%) showed treatment failure. As shown in Table 4, the common type of treatment failure was late parasitological failure, which accounted for 55% of all treatment failures. Based on the per protocol analysis, the cure rate using AL was 98.2% (PCR corrected) and 97.01% (PCR uncorrected). This shows that the reinfection rate within 28 days was 1.1% and the recrudescence rate was 1.9%. Reinfection is the development of malarial signs and symptoms due to a new strain, while recrudescence indicates that the infection has recurred from persistent blood stages of the malaria parasite (Additional file 2).

**Table 4:** Treatment outcome

Outcome ^a^			*n* (%)
Treatment failure		ETF *n* (%)	3 (0.25%)
		LPF *n* (%)	15(1.27%)
		LCF *n* (%)	9 (0.76%)
Adequate response		ACPR	95.5%
Cure rate	Per protocol analysis	PCR unadjusted (%)	97.2%
		PCR adjusted (%)	98.2%
	Intension to treat analysis	PCR unadjusted (%)	92.04%
		PCR adjusted (%)	92.98%

^a^this is reported based on 28 days follow up outcome for all the studies

**Fig. 2:**
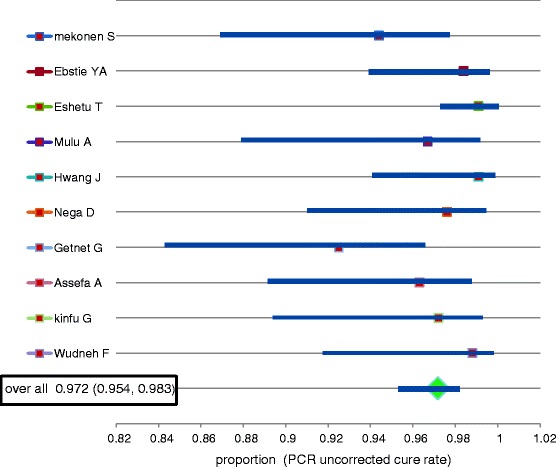
Forest plot for the PCR uncorrected cure rate based on the per protocol analysis

**Fig. 3:**
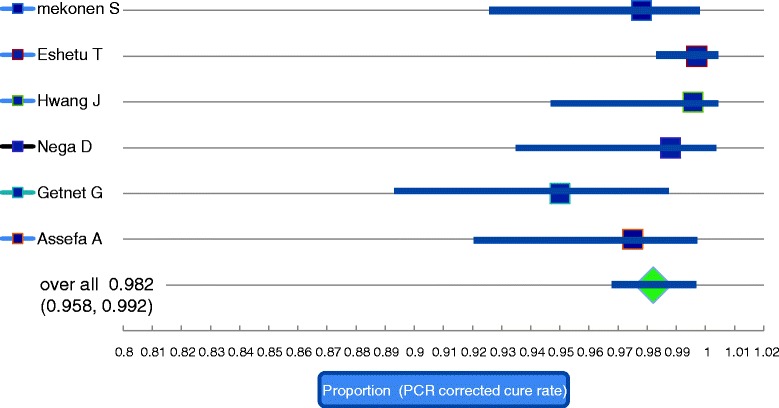
Forest plot for the PCR corrected cure rate based on the per protocol analysis

Heterogeneity between the studies was minimal (I^^2^ = 38.8). As shown in the forest plots provided in Figs. 2 and 3, the 95% confidence interval was overlapping and there was no any outlier study i.e. there is no data value that differ greatly from the majority of data sets (cure rate). The PCR corrected and PCR uncorrected overall cure rates (summary effect) of AL were 0.982 and 0.972, respectively.

## Discussion

AL has been the first-line drug used in Ethiopia for the treatment of uncomplicated falciparum malaria since 2004 after the development of resistance for older antimalarials such as chloroquine and sulphadoxine-pyrimethamine [37]. Since its introduction, no significant AL-resistant *P. falciparum* cases have been reported in the country [17, 31, 32, 38]. The adequate clinical and parasitological responses observed in this analysis similarly show a high efficacy of AL against uncomplicated *P. falciparum* malaria in Ethiopia.

The mean temperature of patients on the day of enrolment was 38.2 ± 0.35 °C. Closer mean temperature at day zero was reported in similar studies conducted in Sudan (38.5 ± 0.6 °C) [39] and Zambia (38.7 °C) [40]. The fever clearance rate of AL was rapid. Fever is one of the common manifestations of malaria that frequently causes discomfort. Thus, it was encouraging that more than 95% of patients treated with AL become fever-free within the first two to three days. Administration of an antipyretic (paracetamol) for febrile patients (body temperature above 38 °C) is common practice. The use of AL for treatment of uncomplicated *P. falciparum* malaria reduces the number of patients who require paracetamol during the follow-up period, which means that the number of drugs taken by patients and thus the risks associated with them is reduced. Other studies also confirm the rapid fever clearance capacity of AL [41–43]. This inherent nature of AL of being able to rapidly reduce or resolve clinical symptoms makes it attractive for patients.

From the 1179 patients included in the 10 studies, 1161 (98.5%) had parasitic clearance within the first three days of treatment with AL. This rate of parasite clearance was consistent with other reports [44–47]. In addition to the rapid parasite clearance, there was also a significant decrease in gametocyte carriage, from 7.7% at baseline to 0.4% on the 28th day of treatment. As confirmed by other studies, artemisinin-based combination therapy is known for its rapid parasite clearance rate and for reducing gametocyte carriage. The low gametocyte carriage after treatment reduces transmission of gametocytes to mosquitoes. Therefore, AL would not only reduce the problem of resistant malaria but also lessen the chances that an infected person might transmit the infection to mosquitoes and to other members of the community [48, 49].Previous studies have reported that artemisinin derivatives are gametocidal [50, 51].The very small proportion of patients with gametocytes at day 28, as found from reviewing 10 studies in this report, supports the results obtained by Targett et al., who reported that an artemisinin derivative has an effect on the sexual stage of the parasite and that specifically AL can decrease gametocytes by 6–8 fold as compared to chloroquine and sulphadoxine-pyrimethamine [51].

In contrast to the finding of the current review, a slower parasite clearance rate and increased third day parasitemia was reported in studies conducted on the Thai-Cambodian border [23, 24].The higher third day parasitic clearance rate (1.5% parasite positivity) reported in the present study may indicate less probability of AL resistance, as Stepniewska et al. explained that resistance is unlikely if the proportion of day three parasite positive smear is less than 3% [52].

The PCR corrected cure rate of AL in the current review was found to be 98.2%. This reveals that AL is highly effective in the treatment of uncomplicated *P. falciparum* malaria. Similar findings were reported by other studies conducted in different parts of the world [16, 53–55]. Studies conducted in East African countries also report a high efficacy of AL [56–59]. A meta-analysis published in 2009 that addressed 32 randomized studies on efficacy of AL reported a 28-day PCR corrected parasitological cure rate of 97% [60]. A community-based study conducted in three African countries (including Ethiopia) also reported that AL was effective in reducing the risk of malaria-specific mortality by 37% [25].

The WHO recommends that for an antimalarial drug to be selected as a first-line treatment agent, it should have a clinical and parasitological cure rate of 90% or higher [15]. The result of the current review is in agreement with the WHO requirement to support the first-line use of AL in the treatment of uncomplicated *P. falciparum* malaria. Therefore, in Ethiopia AL can still be used as a first-line agent in the treatment of uncomplicated *P. falciparum* malaria. This will not, however, guarantee the avoidance of subsequent timely monitoring for its efficacy. The WHO has said that the efficacy of first-line medicines must be cheeked at least every two years [15].

The follow-up period in most of the studies reviewed was 28 days. Two of the studies followed patients for 42 days and one of them reported that the rate of recrudescence was higher at day 42 than at day 28 (PCR corrected cure rate of 94.3% at day 42 and 99.7% at day 28 using per protocol analysis) [30]. This indicates that treatment failure may occur even after longer periods. Therefore, studies that measure the efficacy of AL beyond one month (28 days) are recommended.

In all of the 10 studies reviewed, drug levels were not tested, therefore it is difficult to attribute treatment failure to the drug’s ineffectiveness or development of resistance, as it may be due to insufficient drug levels. It is thus better if future studies conducted on AL efficacy measure the serum drug levels and correlate these with the outcome. Sensitivity of PCR genotyping may have also affected the results of this review, as similar strains, defined as recrudescence, could be new infections, especially in low to moderate transmission areas with a limited diversity of strains [61].

Even though the morning doses of AL were taken in front of investigators in most of the studies, the night time intake of the drug was not directly controlled. Therefore, it was difficult to determine whether some of the treatment failure cases reported were due to low adherence. There are also many other factors that may influence the treatment outcome. Further studies that identify risk factors for treatment failure should be conducted. Studies that describe in detail the characteristics of patients with recrudescence are also very important. Molecular surveillance may also play an important role in detecting genetic markers associated with AL resistance in the local *P. falciparum* population.

## Conclusions

AL is highly efficacious in Ethiopia for the treatment of uncomplicated *P. falciparum* malaria even after a number of years of its widespread use in the country. There should, however, be careful periodic monitoring of the efficacy of this drug, as treatment failure may occur due to resistance, sub-therapeutic levels that may occur due to non-adherence, or inadequate absorption.

## Additional files

Additional file 1: Multilingual abstracts in the five official working languages of the United Nations. (PDF 799 kb)

Additional file 2: Treatment outcome from individual study. (DOCX 18 kb)

## Abbreviations

AL: artemether-lumefantrine
NIH: National Institute of Health
PCR: polymerase chain reaction
WHO: World Health Organization
