# Supplementary material for: Therapeutic efficacy of artemether-lumefantrine in the treatment of uncomplicated Plasmodium falciparum malaria in Ethiopia: a systematic review and meta-analysis
Source: Infect Dis Poverty. 2017 Nov 15;6:157. doi: 10.1186/s40249-017-0372-5 (PMC5686809; doi:10.1186/s40249-017-0372-5)
Supplement: Supplementary file 1 — Multilingual abstracts in the five official working languages of the United Nations. (PDF 799 kb) [file 40249_2017_372_MOESM1_ESM.pdf]

Translation of the abstract into the five official working languages of the United Nations

الفعالية العلاجية لمادة أرتيميثير-لومفانترين في المعالجة غير المصحوبة بمضاعفات الملاريا المنجلية في إثيوبيا: مراجعة منهجية وتحليل تجميعي

محمد بيسيت أيالو

ملخص

الهدف: كان الهدف من هذه الدراسة هو تجميع الأدلة المتاحة على فعالية مادة أرتيميثير-لومفانترين في المعالجة غير المصحوبة بمضاعفات الملاريا المنجلية في إثيوبيا. وقد تم ذلك عن طريق إجراء تحليل تجميعي للدراسات الأخيرة التي أجريت في البلد بشأن هذا الموضوع.

الأساليب: بحثت الدراسات المنشورة بين يناير 2010، ويناير 2017 التي أبلغت عن مدى فعالية مادة أرتيميثير-لومفانترين في علاج الملاريا المنجلية في المرضى الإثيوبيين باستخدام قاعدة بيانات PubMed ومحرك جوجل. شمل التحليل الدراسات الحشدية الاستباقية العشر ذات الفرع الواحد التي تابعت المرضى لمدة 28-42 يومًا. واعتبرت كل من الدراسات المشمولة ذات جودة عالية.

النتائج: حددت عشر دراسات شملت 1179 مريض والتي كانت مؤهلة للتحليل التجميعي. عند التعيين، كان المعدل الطفيلي المتوسط للمريض الواحد 12 981/ميكرو ليتر من الدم. في اليوم الثالث من العلاج، أصبح 96.7 في المائة و 98.5 في المائة من مواضيع الدراسة خالية من الحمى وخالية من الطفيليات، على التوالي. استنادًا إلى تحليل كل البروتوكول، كانت نسبة الشفاء بعد استخدام أرتيميثر-لوميفانترين 98.2٪ (تفاعل بوليميراز متسلسل مصحح) و 97.01٪ (تفاعل بوليميراز متسلسل غير مصحح) بعد 28 يومًا من المتابعة. وكان معدل عودة العدوى خلال 28 يوما 1.1٪ ومعدل عودة المرض 1.9٪.

الاستنتاجات: وجد هذا الاستعراض أن نسبة الشفاء للملاريا غير المعقدة المنجلية باستخدام أرتيميثر-لوميفانترين في إثيوبيا لا تزال مرتفعة بما فيه الكفاية للتوصية بالأدوية كعامل أولي. يجب أن يكون هناك رصد دوري دقيق لفعالية هذا الدواء، كما قد يحدث فشل العلاج بسبب المقاومة، أو مستويات دون المستوى العلاجي التي قد تحدث بسبب عدم الالتزام، أو عدم الامتصاص الكافي.

Translated from English version into Arabic by Eman Shahen, through

TRANSLATORS
WITHOUT BORDERS

在埃塞俄比亚使用蒿甲醚-本芴醇治疗单纯性恶性疟的疗效：一项系统性回顾研究和荟萃分析

Mohammed Biset Ayalew

摘要

引言：本研究的目的是综合收集在埃塞俄比亚使用蒿甲醚-本芴醇治疗单纯性恶性疟的疗效证据，对该国近期这一专题研究进行荟萃分析。

方法：使用 PubMed 和谷歌学术数据库搜索 2010 年 1 月-2017 年 1 月之间发表的在埃塞俄比亚使用蒿甲醚-本芴醇治疗恶性疟患者的疗效的研究报告。这项分析包括 10 项前瞻性单臂队列研究，随访时间在 28～42 d。所有纳入的研究均被认为是高质量的。

结果：确定 10 项研究中的 1 179 例患者符合 meta 分析要求。在募集时，平均每例原虫计数为 12 981 /µl 血。治疗第 3 天，96.7%和 98.5%的病例分别表现为无发热和无寄生虫。基于每

项拟定分析，经过 28 d 的随访，使用蒿甲醚-本芴醇的治愈率为 98.2%（PCR 鉴定校正）和 97.01%（PCR 鉴定未校正）。28 d 内再感染率为 1.1%，复发率为 1.9%。
结论：研究发现，在埃塞俄比亚使用蒿甲醚-本芴醇治疗单纯性恶性疟的治愈率很高，可推荐该药物作为一线药物。治疗失败可能是由于耐药性，或患者依从性不好或吸收不足而引起的亚治疗水平，因此，应谨慎定期监测该药物组合的疗效。

Translated from English version into Chinese by Zhou Guan, edited by Pin Yang

CHINESE CENTER FOR DISEASE CONTROL AND PREVENTION
NATIONAL INSTITUTE OF PARASITIC DISEASES

L'efficacité thérapeutique de l'artéméther-luméfantrine dans le traitement de la malaria *Plasmodium falciparum* en Éthiopie : revue systématique et méta-analyse

Mohammed Biset Ayalew

Résumé
Objectif : le but de cette étude est de synthétiser les données disponibles sur l'efficacité de l'artéméther-luméfantrine dans le traitement de la malaria *Plasmodium falciparum* non compliquée en Éthiopie. Cela a été fait en procédant à une méta-analyse des études récentes effectuées dans le pays sur ce sujet.
Méthodes : les études publiées entre janvier 2010 et janvier 2017 qui ont rapporté l'efficacité de l'artéméther-luméfantrine dans le traitement de la malaria *P. falciparum* chez les patients éthiopiens ont été recherchées en utilisant les bases de données PubMed et Google Scholar. Dix études de cohortes prospectives d'une branche, qui ont suivi les patients pendant 28 à 42 jours, ont été inclues dans cette analyse. Toutes les études inclues sont réputées de grande qualité.
Résultats : dix études portant sur 1 179 patients qui remplissaient les conditions pour la méta-analyse ont été identifiées. Lors du recrutement, le nombre de parasites moyen par patient était de 12 981/µL de sang. Le troisième jour de traitement, 96,7 % et 98,5 % des sujets de l'étude étaient respectivement exempts de fièvre et de parasite. Basé sur l'analyse per protocole, le taux de guérison après utilisation de l'artéméther-luméfantrine était de 98,2 % (réaction en chaîne par polymérase, corrigé) et de 97,01 % (réaction en chaîne par polymérase, non corrigé) après 28 jours de suivi. Le taux de réinfection dans les 28 jours était de 1,1 % et le taux de recrudescence de 1,9 %.
Conclusions : Cette analyse a montré que le taux de guérison pour la malaria *P. falciparum* non compliquée à l'aide de l'artéméther-luméfantrine en Éthiopie est encore assez élevé pour recommander ce médicament comme traitement de première intention. Il doit y avoir un suivi périodique attentif de l'efficacité de ce médicament, car un échec du traitement peut se produire en raison de la résistance, des taux sous-thérapeutiques qui pourraient découler de la non-observance ou d'une absorption inadéquate.

Translated from English version into French by Julie Thonus, through

Терапевтическая эффективность артеметер-люмефантрина при лечении неосложненной малярии *Plasmodium falciparum* в Эфиопии: системный обзор и мета-анализ

Мохаммед Бисет Айалев

Выдержка

Цель: Цель данного исследования состояла в обобщении имеющихся данных об эффективности артеметер-люмефантрина при лечении неосложненной малярии, вызванной*Plasmodium falciparum*, в Эфиопии. В основу был положен мета-анализ последних исследований на эту тему, проведенных в стране.

Методы: Был проведен анализ исследований, опубликованных в период с января 2010 по январь 2017 г., в которых сообщалось об эффективности артеметер-люмефантрина при лечении малярии, вызванной *P. falciparum*, на использование баз данных PubMed и Google Scholar. В данный анализ были включены результаты десяти проспективных несравнительных когортных исследований, в которых за пациентами осуществлялось наблюдение на протяжении 28 – 42 дней. Подразумевается, что все включенные в анализ исследования, были высокого качества.

Результаты: </bx>Были рассмотрены десять исследований, в которых были задействованы 1 179 пациентов, удовлетворяющие параметрам мета-анализа. При отборе, средний показатель количества паразитов на пациента составлял 12 981/µL крови. На третий день лечения 96,7% и 98,5% участников исследования были избавлены от лихорадки и паразитов, соответственно. На основании анализа в соответствии с протоколом, частота выздоровлений после использования артеметер-люмефантрина составила 98,2% (полимеразная цепная реакция скорректирована) и 97,01% (полимеразная цепная реакция не скорректирована) по истечении 28 дней наблюдения. Через 28 дней частота повторного инфицирования составила 1,1%, а частота рецидивов – 1,9%.

Выводы: Данное исследование продемонстрировало, что частота выздоровлений от неосложненной малярии, вызванной возбудителем *P. falciparum* , при применении артеметер-люмефантрина в Эфиопии, все еще находится на высоком уровне, что позволяет рекомендовать данное лекарство в качестве препарата первого ряда. Следует производить тщательный периодический контроль эффективности данного лекарственного средства, поскольку безрезультатность терапии может быть обусловлена невосприимчивостью, субтерапевтическими уровнями, которые могут возникать в результате несоблюдения назначений или неполноценной абсорбции.

Eficacia terapéutica de arteméter y lumefantrina en el tratamiento de la malaria no complicada producida por *Plasmodium falciparum* en Etiopía: revisión sistemática y metaanálisis

Mohammed Biset Ayalew

Resumen

Objetivo: El objetivo de este estudio es compendiar la evidencia disponible sobre la eficacia de arteméter y lumefantrina en el tratamiento de la malaria no complicada producida por *Plasmodium falciparum* en Etiopía. Esto se ha llevado a cabo mediante un metaanálisis de estudios recientes realizados en el país sobre este tema.

Métodos: Se hizo una búsqueda de los estudios publicados entre enero de 2010 y enero de 2017 que informaban sobre la eficacia de arteméter y lumefantrina en el tratamiento de pacientes etíopes de malaria producida por *P. falciparum* utilizando las bases de datos PubMed y Google Scholar. Se incluyeron en este análisis diez estudios prospectivos de cohorte de brazo único que realizaron un seguimiento de los pacientes durante un periodo de entre 28 y 42 días. Todos los estudios incluidos se consideraron de alta calidad.

Resultados: Se identificaron diez estudios que implicaban a 1 179 pacientes y que eran elegibles para metaanálisis. En el momento de ser reclutados, el promedio de contaje de parásitos por paciente fue de 12 981/µl de sangre. Al tercer día de tratamiento, el 96,7 % y el 98,5 % de los sujetos del estudio estaban libres de fiebre y libres de parásitos, respectivamente. Basándose en el análisis por protocolo, la tasa de curación después del uso de arteméter y lumefantrina fue de 98,2 % (corregido con la reacción en cadena de la polimerasa) y 97,01 % (sin corregir con la reacción en cadena de la polimerasa) después de 28 días de seguimiento. La tasa de reinfección en los primeros 28 días fue del 1,1 % y la tasa de recrudecimiento fue del 1,9 %.

Conclusiones: Esta revisión concluye que la tasa de curación para la malaria no complicada producida por *P. falciparum* en Etiopía usando arteméter y lumefantrina sigue siendo lo suficientemente alta como para recomendar el fármaco como agente de primera línea. Debería realizarse un meticuloso control periódico de la eficacia de este fármaco, ya que se puede producir un fracaso del tratamiento debido a la resistencia, niveles subterapéuticos pueden ocurrir debido a la falta de adherencia o absorción inadecuada.

Translated from English version into Spanish by mariagloria02, through
