# Supplementary material for: Therapeutic efficacy of artemether-lumefantrine in the treatment of uncomplicated Plasmodium falciparum malaria in Ethiopia: a systematic review and meta-analysis
Source: Infect Dis Poverty. 2017 Nov 15;6:157. doi: 10.1186/s40249-017-0372-5 (PMC5686809; doi:10.1186/s40249-017-0372-5)
Supplement: Supplementary file 2 — Treatment outcome from individual study. (DOCX 18 kb) [file 40249_2017_372_MOESM2_ESM.docx]

Treatment outcome from individual study

| **Sr.no.** | **Author**  **(year of publication)** | **Fever clearance** | | | **Parasite Clearance** | | | **Gametocyte carriage** | **Treatment outcome** | | | | **Cure rate**  ITT analysis | | Cure rate  **PP analysis** | |
| --- | --- | --- | --- | --- | --- | --- | --- | --- | --- | --- | --- | --- | --- | --- | --- | --- |
|  |  | Day 1 | Day 2 | Day 3 | Day 1 | Day 2 | Day 3 | Day 28 | ETF | LPF | LCF | ACPR | PCR Un corrected cure rate | PCR corrected cure rates | PCR Un corrected cure rate | PCR corrected cure rates |
| 1 | Mekonnen SK. et al. (2015) | 83.1% | 94.4% | 100% | 88.8  % | 96.6% | 100% | 2 (2.2%) | 0 | 4 (4.5 %) | 1(1.1 %) | 94.4 % | 94.4 % (95 % CI 88.0–97.9 %) | 97.8 % (95  % CI 92.8–99.6  %) | 94.4 % (95 % CI 88.0–97.9 %) | 97.8  % (95  % CI 92.8–99.6  %) |
| 2 | Ebstie YA. et al. (2015) | NR | NR | 87.8% | NR | 85.9% | 96.1 % | 0 | 0 | 2 (1.56%) | 0 | 98.4% | 95.5% | NR | 98.4% | NR |
| 3 | Eshetu T. et al. (2012) | NR | 96.7% | 91.9% | NR | 98.2% | 99.4% | Day 28^th^  3(1%)  Day 42^nd^  2(0.7%) | 0 | Day 28^th^ 0  Day 42^nd^  12 (3.4) | Day 28^th^ 3(0.9)  Day 42^nd^  16(4.6) | Day 28^th^ 89.7%  Day 42^nd^ 82.2% | Day 28^th^  89.7%, (86.4-92.9)  Day 42^nd^  82.2, (78.1-86.2) | Day 28^th^  312(89.9, 86.7-93.1)  Day 42^nd^  85.1, (81.3-88.9) | Day 28^th^  99.1% (95%  CI 98.0-100.0)  Day 42^nd^  91.1% (95% CI 87.9-94.3) | Day 28^th^  99.7%  Day 42^nd^  (94.3%) |
| 4 | Mulu A. etal (2015) | 89.4% | 98.5% | 100% | 84.8% | 93.9% | 96.8% | NR | 1(1.64%) | 1(1.64%) | 0 | 96.7% | 90.9% | NR | 96.7% | NR |
| 5 | Hwang J. et al. (2011) | 65.2% | 91.5% | 93% | NR | 93.1% | 99.1% | 0 | 0 | 1(on day 28)  1(on day 42) | 0 | 28th Day 93.3%  42^nd^ day 86.6% | 28th Day  93.3% (87.2-97.1)    42^nd^ day  86.6% (79.1-92.1) | 28th Day  94.1% (88.2-97.6)  42^nd^ day  87.3% (79.9-92.7) | Day 28 99.1%  (95% CI 95.1-100.0)  Day 42= 99.0% (95% CI 94.8-100.0 | day 28  100.0% (95% CI 96.7-100.0) day 42 100.0% (95% CI  96.5-100.0) |
| 6 | Nega D et al. (2016) | 78.7% | 94.3% | 97.7% | 69.7% | 95.5% | 100% | 0 | 0 | 1(1.2%) | 1(1.2%) | 97.6% (95%CI: 93.6–99.5) | 91.2% (95%CI: 85.6–93.1%) | 92.2% (95%CI: 86.2–96.1) | 97.6% (95%CI: 93.6–99.5) | 98.8% (95%CI: 93.5–100%) |
| 7 | Getnet G.  et al. (2015) | 75% | 91.3% | 96.2% | 73.8% | 91.3% | 94.9% | 0 | 2 (2.5%) | 3 (3.75%) | 1 (1.25%) | 74 (92.5%) | 92.5% | 95.0% (95% CI 87.0–98.4%) | 92.5% | 95.0% (95% CI 87.0–98.4%) |
| 8 | Assefa A. et al. (2010) |  |  | 100% |  |  | 98.9% | 0 | 0 | 2(2.4%) | 1(1.2%) | 79 (96.3%) | 87.8% | 88.9% | 96.3% 95% CI: 89.7-99.2) | 97.5% |
| 9 | Kinfu G. et al (2012) |  | 100% | 100% |  | 100% | 100% | 0 | 0 | 0 | 2(2.8%) | 69 (97.2%) | 97.2% | NR | 97.2% | NR |
| 10 | Wudneh F. et al (2016) | 69.6% | 97.8% | 100% | 23.6% | 91.0% | 100% | 0 | 0 | 1(1.2%) | 0 | 80(98.8) | 87.9% | NR | 98.8% | NR |
